# Genomic characteristics and comparative genomics of *Salmonella enterica* subsp. *enterica* serovar Schwarzengrund strain S16 isolated from chicken feces

**DOI:** 10.1186/s13099-021-00476-8

**Published:** 2022-01-04

**Authors:** Seung-Min Yang, Eiseul Kim, Woojung Lee, Hae-Yeong Kim

**Affiliations:** Institute of Life Sciences & Resources and Department of Food Science and Biotechnology, Kyung Hee University, Yongin, 17104 South Korea

**Keywords:** *Salmonella*, Schwarzengrund, Whole-genome sequencing, Comparative genomics, Antibiotic resistance gene, Virulence gene

## Abstract

**Background:**

*Salmonella enterica* subsp. *enterica* serovar Schwarzengrund (*S*. Schwarzengrund) is most frequently isolated from commensals humans or poultry. Here we report *S*. Schwarzengrund strain S16, the first sequenced genome in the Republic of Korea. Additionally, genome sequencing for strain S16 was performed and compared with other *S*. Schwarzengrund genomes obtained from public database.

**Results:**

Strain S16 was isolated from chicken feces. The complete genome consists of one chromosome and one plasmid. The genome size is 4,822,755 bp with 4852 coding sequences. Strain S16 was determined as serovar Schwarzengrund by in silico serotyping and typed as sequence type (ST) 96. Forty-six *S*. Schwarzengrund genomes yielded a pangenome of 7112 genes, core-genome of 3374 genes, accessory-genome of 2906 genes, and unique-genome of 835 genes. Eighty-one genes were unique to strain S16, including hypothetical proteins and transcriptional regulators. Genotypic analysis of antibiotic resistance of strain S16 confirmed resistance to amikacin, ciprofloxacin, sulfamethoxazole, streptomycin, and tetracycline. Unlike other *S*. Schwarzengrund genomes, strain S16 had a mutation of *gyrB*. Moreover, similar to other *S*. Schwarzengrund genomes reported in other countries, strain S16 was harbored for 153 virulence genes including *Saf* operon and *cdtB* gene. All the antibiotic resistance genes and virulence genes were present in the core- or accessory-genomes.

**Conclusions:**

Complete genome of strain S16 was sequenced. Comparative genomic analysis revealed several genes responsible for antibiotic resistance and specific genomic features of strain S16 and identified virulence factors that might contribute to the human and animal pathogenicity of other *S*. Schwarzengrund genomes.

**Supplementary Information:**

The online version contains supplementary material available at 10.1186/s13099-021-00476-8.

## Background

*Salmonella* is a major human pathogen and is the second most prevalent foodborne pathogen worldwide [1]. Salmonellosis, a disease caused by *Salmonella*, represents a severe global public health problem, leading to mortality and morbidity and resulting in a high socioeconomic burden [2]. *Salmonella* is represented by more than 2600 serovars. *Salmonella enterica* subsp. *enterica* serovar Schwarzengrund (*S.* Schwarzengrund), a *Salmonella* serovar, infects humans and poultry in several countries [3]. According to a previous study, in many countries, *S*. Schwarzengrund is one of the top 15 *Salmonella* serovars isolated from humans, foods, and animals [4]. In Korea, it ranks one of the top 25 *Salmonella* serovars identified in humans, foods, and animals over the past decade [5]. Previously, *S.* Schwarzengrund infection occurred in multiple states in the United States, and an outbreak investigation revealed that contaminated dry dog food was the cause [6]. This serotype has increased the prevalence and proportion of human infection caused by *S*. Schwarzengrund in Asia, the United States, Denmark, and Brazil, and multidrug resistance has been exhibited by several isolates [3, 7–9].

Advances in whole-genome sequencing (WGS) technology have become an economically viable alternative to conventional typing methods for outbreak investigation and public health surveillance [1]. Comparative genomics with WGS data provides insight into the genome of pathogenic bacteria, including candidate drug compounds, potential virulence determinants, mechanisms of pathogenicity, and their evolution in pathogens. As a result, this technology has been instrumental in improving diagnostics and public health microbiology [10]. Currently, this technique is widely used to investigate the outbreak of pathogenic bacteria, such as *Bacillus cereus*, *Escherichia coli*, *Vibrio parahaemolyticus*, and *Salmonella* species [11–14].

Although *S*. Schwarzengrund is one of the most frequently isolated serovars from livestock, such as cattle, pigs, and chickens, in the Republic of Korea [15], the genome sequence of the *S*. Schwarzengrund strain isolated in Korea has not been reported. This is the first study in the Republic of Korea to report the complete genome sequence of *S*. Schwarzengrund strain S16, a pathogenic bacterium with antibiotic resistance and virulence factors, isolated from chicken feces. Furthermore, we accomplished functional-level genomic comparison with the previously reported *S*. Schwarzengrund strains.

## Methods

### Bacterial strain isolation

The strain S16 was isolated from a fecal sample of chicken. First, 25 g of fecal sample was enriched in 225 ml of buffered peptone water (BPW) (Difco, Becton Dickinson, Sparks, MD, USA) at 37 °C for 18 h. Next, an aliquot of 0.1 ml BPW suspension was transferred into a 10 ml Rappaport–Vassiliadis broth (Difco) and cultured at 42 °C for 24 h. Then, a loopful was streaked on xylose lysine deoxycholate (XLD) agar (Difco) and incubated at 37 °C for 24 h. Colonies exhibiting typical *Salmonella* morphology (red colonies with black centers, black-colored colonies) were confirmed using matrix-assisted laser desorption ionization time-of-flight mass spectrometry (Bruker Daltonics, Bremen, Germany).

### Genome sequencing, assembly, and annotation

The genomic DNA was extracted using DNeasy blood and tissue kit (Qiagen, Hilden, Germany) according to the manufacturer’s protocol. The library was prepared using SMRTbell template prep kit v.1.0 (Pacific Biosciences, Menlo Park, CA, US). The complete genome sequencing was performed using PacBio Sequel IIe (Pacific Biosciences) sequencer using Sequel sequencing kit v.3.0. Raw reads were processed using demultiplex barcode protocol, distributed with the SMRT Link v.10.1 to remove the index and raw quality reads with default parameters. De novo assembly was performed using microbial assembly protocol, which is part of PacBio SMRT Link. The assembled genome was annotated by Rapid Annotation using Subsystem Technology server v.2.0.

The wgVISTA program (https://genome.lbl.gov/cgi-bin/WGVistaInput) was used to compare the reference genome with strain S16 genome. The reference genome was used as *S*. Schwarzengrund CVM19633 (accession no. CP001127.1). Incompatibility (Inc) type of plasmid was identified using PlasmidFinder v.2.0 [16]. Putative prophage element in the strain S16 genome was identified using PHASTER tool [17]. Insertion sequence (IS) in the strain S16 genome was identified using ISfinder [18], and transposon was detected using TnFinder [19]. The genomic island was analyzed using IslandViewer 4 [20].

### Taxonomy classification

Average nucleotide identity (ANI) based on WGS was used to determine the subspecies of strain S16. ANI value was calculated using OrthoANI [21]. The serotype of strain S16 was predicted using SeqSero 2.0 and *Salmonella *In Silico Typing Resource (SISTR) [22, 23]. The SeqSero 2.0 command-line tool was obtained from Github (https://github.com/denglab/SeqSero2). The assembled genome was directly uploaded to the SISTR website for serovar prediction. Multi-locus sequence type (MLST) was analyzed using the PubMLST database (https://pubmlst.org/).

### Antimicrobial susceptibility testing for strain S16

The minimum inhibitory concentrations (MICs) for 21 antimicrobial agents were evaluated by broth microdilution assay using Sensititre KRCDC2F and KRNV5F plates (TREK Diagnostic Systems, West Sussex, UK), according to the manufacturer’s instructions. Seventeen antimicrobial agents used in this study were as follows: amikacin (AMK), gentamycin (GEN), streptomycin (STR), ampicillin (AMP), amoxicillin/clavulanic acid (AUG2), cefoxitin (FOX), ceftiofur (XNL), ceftazidime (CAZ), cefepime (FEP), meropenem (MEM), ciprofloxacin (CIP), trimethoprim/sulfamethoxazole (SXT), sulfisoxazole (FIS), chloramphenicol (CHL), colistin (COL), imipenem (IMI), cefotaxime (FOT), ceftriaxone (AXO), azithromycin (AZI), nalidixic acid (NAL), and tetracycline (TET). The breakpoint interpretation of antimicrobial agents was determined according to the Clinical and Laboratory Standards Institute guidelines [24].

### Comparative genomic analysis

A comparative genomic analysis was performed among the *S*. Schwarzengrund S16 and 45 other strains of *S*. Schwarzengrund. Forty-five genomes assembled at the complete and scaffold level were downloaded from the National Center for Biotechnology Information (NCBI, Accessed on August 06, 2021) for comparative genomic analysis. Information such as origin, geographic, collection date, and host of the 46 genomes used in this study is shown in Additional file 1: Table S1. Pangenome analysis was performed using the bacterial pan genome analysis tool (BPGA) [25]. Phylogenetic tree based on concatenated core gene alignment was constructed using MUSCLE, a built-in BPGA tool with 1000 bootstrap replicates. The functional genes were predicted using the USEARCH program against the cluster of orthologous groups of proteins (COGs) database within the BPGA pipeline, with the default parameters. Virulence factors and antimicrobial resistance genes were identified using the virulence factor database and ResFinder v.4.1, respectively [26, 27].

### Quality assurance

A single colony of strain S16 was transferred three times in XLD medium to obtain a single pure colony. After complete genome sequencing, the 16S rRNA gene was extracted and identified using the basic local alignment search tool (BLAST) against the NCBI nucleotide collection database. As a result, strain S16 was identified as *S*. Schwarzengrund strain WAPHL_SAL-A00527 (accession number CP054901.1) with 100% identity.

## Results and discussion

### Genomic features

**Fig. 1:**
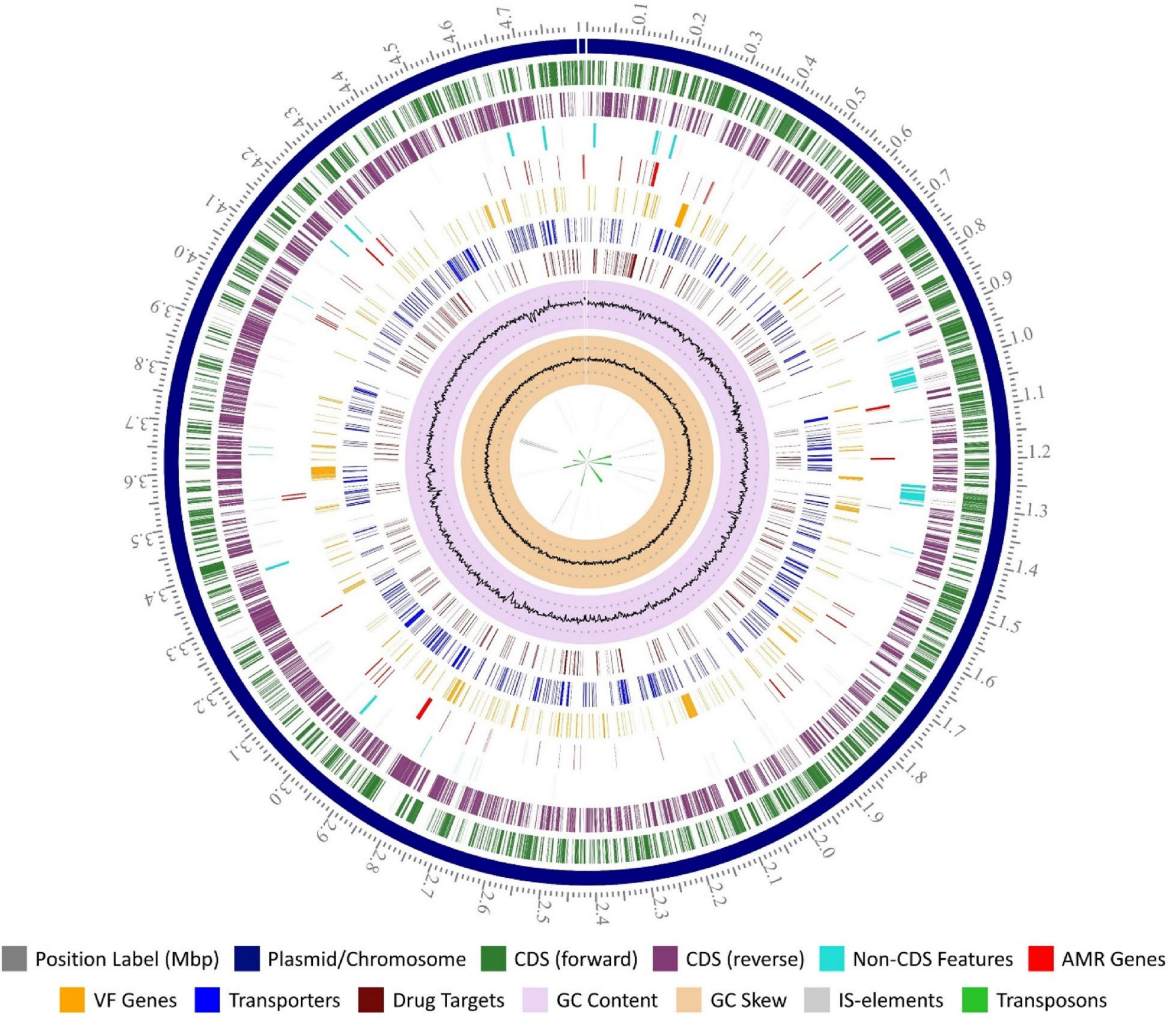
Circular genome mapping of the *S*. Schwarzengrund strain S16. From inner to outer rings, each ring indicates GC skew, GC content, drug target gene, transporter gene, virulence genes, antibiotic resistance genes, non-CDS, reverse CDS, and forward CDS

The strain S16 has a chromosome and one plasmid (pS16) and a total length of 4,822,755 bp (Fig. 1), after quality control of 153,596 raw reads with an average read length of 7866 and N50 size of 9870. The genome coverage was found to be 1800-fold. The chromosome was assembled into 4,811,709 bp with a G+C content of 52.17%. The plasmid size was 11,046 bp and had a G+C content of 61.73%. A plasmid (pS16) had an IncQ1 plasmid replicon sequence, and the IncQ1 replicon region (956–1751 bp) showed high homology with the RSF1010 plasmid of *Escherichia coli* (accession no. M28829.1). IncQ1 plasmids are small (10–12 kb) and broad host range [28]. They are generally associated with aminoglycosides, sulfonamides, and tetracycline resistance genes, although other antimicrobial resistance genes have been identified [28]. Similarly, pS16 carried multiple antibiotic resistance genes including *aph(6)-Id*, *aph(3″)-Ib*, *sul2*, and *tet(A)*, associating resistances of aminoglycoside, sulfonamides, and tetracycline. Annotation of this assembly identified 4852 coding sequences (CDSs), 84 tRNAs, and 22 rRNAs. A total of 4852 CDSs were assigned to COG categories, comprising 20 functional categories. Most of the genes were assigned for the general function prediction only (R, 12.11%), amino acid transport and metabolism (E, 9.66%), and carbohydrate transport and metabolism (G, 8.82%) (Additional file 2: Fig. S1).

Prophages are a critical part of the pathogenic bacterial genome that facilitates various properties for bacteria, such as the acquisition of bacterial resistance [29]. In the strain S16 genome, a total of five prophage regions were identified, and prophage regions are shown in Fig. 1. The prophage 1 region extended from 988,320 to 1,049,335 bp with a GC content of 48.25% and was homologous to genes of the phage vB_SosS_Oslo (accession no. NC018279) found in *Salmonella*. The prophage 2, 3, 4, and 5 regions were homologous to genes of the phage SPN3UB (accession no. NC019545), 118970_sal3 (accession no. NC031940), Fels_1 (accession no. NC010391), Fels_2 (accession no. NC010463) found in *Salmonella*, respectively. Moreover, twenty-two different forms of IS-elements and 11 transposons were detected in the strain S16 genome (Fig. 1; Additional file 1: Table S2). Transposon and IS-element are known to play a major role in the dissemination of resistance genes [30]. Alignment of reference genome to strain S16 genome showed complete congruity except for some regions (Additional file 2: Fig. S2). Strain S16 is highly similar to strain CVM19633, but differs in genomic islands and prophages by having unique islands and prophages that are not present in the CVM19633 genome.

### Taxonomy classification

Taxonomic classification by ANI resulted in the inference of subspecies level identification to *S*. *enterica* subsp. *enterica* NCTC 12416^T^ with an ANI of 98.42% for strain S16. The ANI of strain S16 with *Salmonella* species or subspecies, *S*. *bongori* NCTC 12419^T^, *S*. *enterica* subsp. *salamae* NCTC 5773^T^, *S*. *enterica* subsp. *arizonae* NCTC 8297^T^, *S*. *enterica* subsp. *diarizonae* NCTC 10060^T^, *S*. *enterica* subsp. *houtenae* NCTC 10401^T^, *S*. *enterica* subsp. *indica* NCTC 12420^T^ were 89.61%, 96.13%, 93.21%, 95.18%, 95.06%, and 95.74%, respectively. The serovar of strain S16 was determined as Schwarzengrund (4:d:1,7) by SeqSero. Likewise, strain S16 was also determined as Schwarzengrund based on cgMLST by the SISTR software.

Based on the *Salmonella* species MLST database, the sequence type (ST) was recognized. *S*. Schwarzengrund strain S16 was typed as ST96 that is associated with strains isolated from poultry and environmental sources [31]. Previously, *S*. Schwarzengrund ST96 was associated with a carbapenemase-resistant strain isolated from a human in Argentina and extended-spectrum beta-lactamase producing strain from poultry in Brazil [32, 33].

### Comparative genomics

Pangenome analysis showed that 46 genome sequences of *S*. Schwarzengrund had a pangenome of 7115 genes, which was divided into a core-genome of 3374 genes, an accessory-genome of 2906 genes, and a unique-genome of 835 genes. Here, a set of genes shared by all genomes was defined as core-genome, whereas a set of genes partially shared by genomes and a set of genes unique to a single genome were defined as accessory-genome and unique-genome, respectively [11]. Of the 835 unique genes, 81 were identified as unique genes for strain S16, including the ones encoding helix-turn-helix domain-containing protein, IS*3* family transposase, chitinase, helix-turn-helix transcriptional regulator, and hypothetical proteins (Additional file 1: Table S3). Unique genes of strain S16 were assigned in six COG categories: transcription (K); defense mechanisms (V); replication, recombination and repair (L); energy production and conversion (C); inorganic ion transport and metabolism (P); and general function prediction only (R). The functional category, such as transcription (K, 28.17%), was the most enriched among the unique genes of strain S16 (Additional file 2: Fig. S1).

**Fig. 2:**
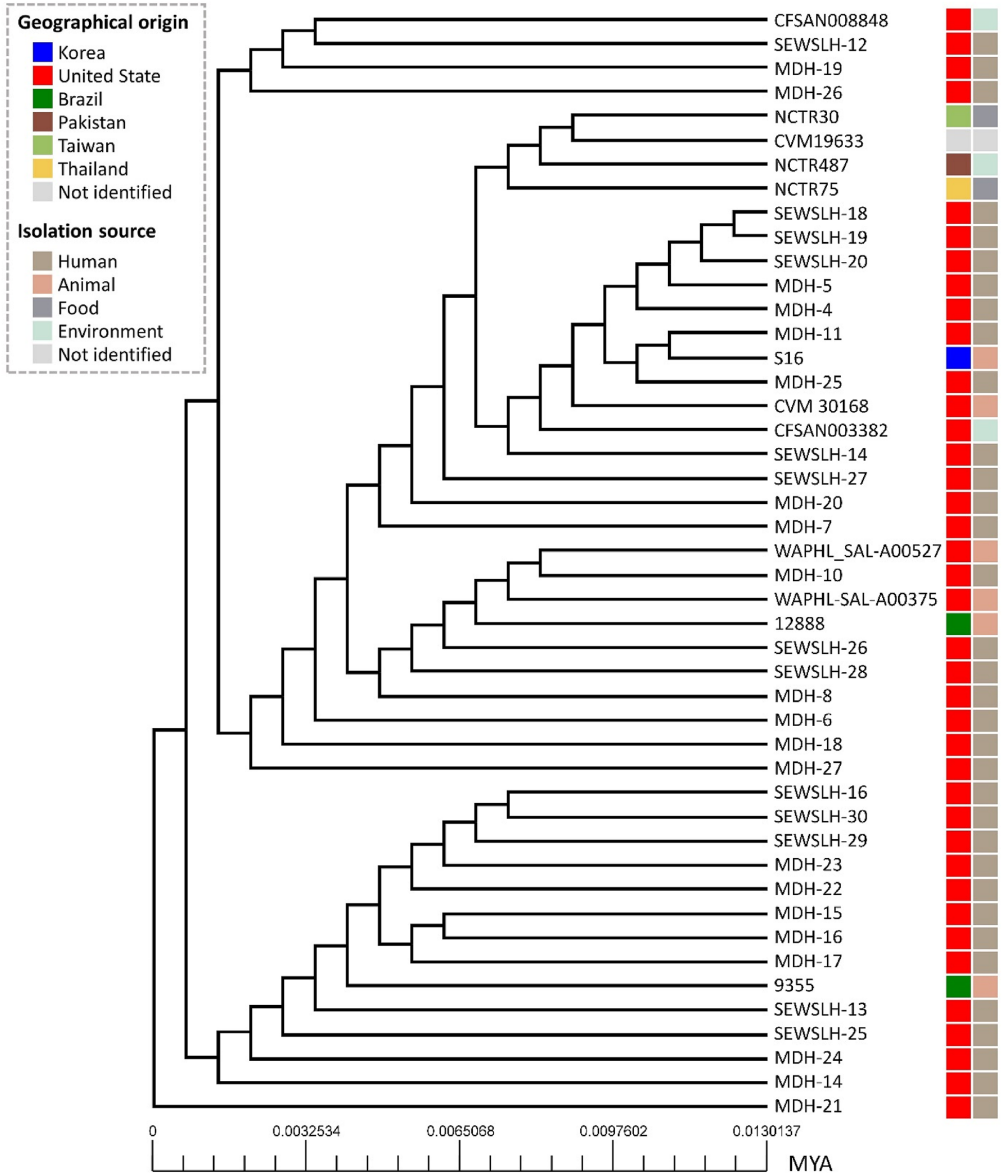
Phylogenetic tree based on concatenated core genes for 46 *S*. Schwarzengrund genomes. The geographical origin and isolation source of strains are color coded. The scale bar is depicted in millions of years (MYA)

To investigate the genetic environment, strain S16 genome was compared with 45 other genomes of *S*. Schwarzengrund. Arrow diagrams for the five genomic islands specific to strain S16 are shown in Additional file 2: Fig. S3). As a result, there were five genomic islands in strain S16 that were not found in 45 other genomes of *S*. Schwarzengrund (Additional file 1: Table S4). Also, the unique genes were not related to mobile genetic element, IS elements, and transposons, but 28 genes out of 81 genes were present on the genomic island (Additional file 1: Table S5). The phylogenetic tree based on core-genome showed that strain S16 clustered with the MDH-25 and MDH-11 genomes (Fig. 2). These strains were isolated from clinical stool samples from humans [8].

### In silico identification of antibiotic-resistance genes

**Fig. 3:**
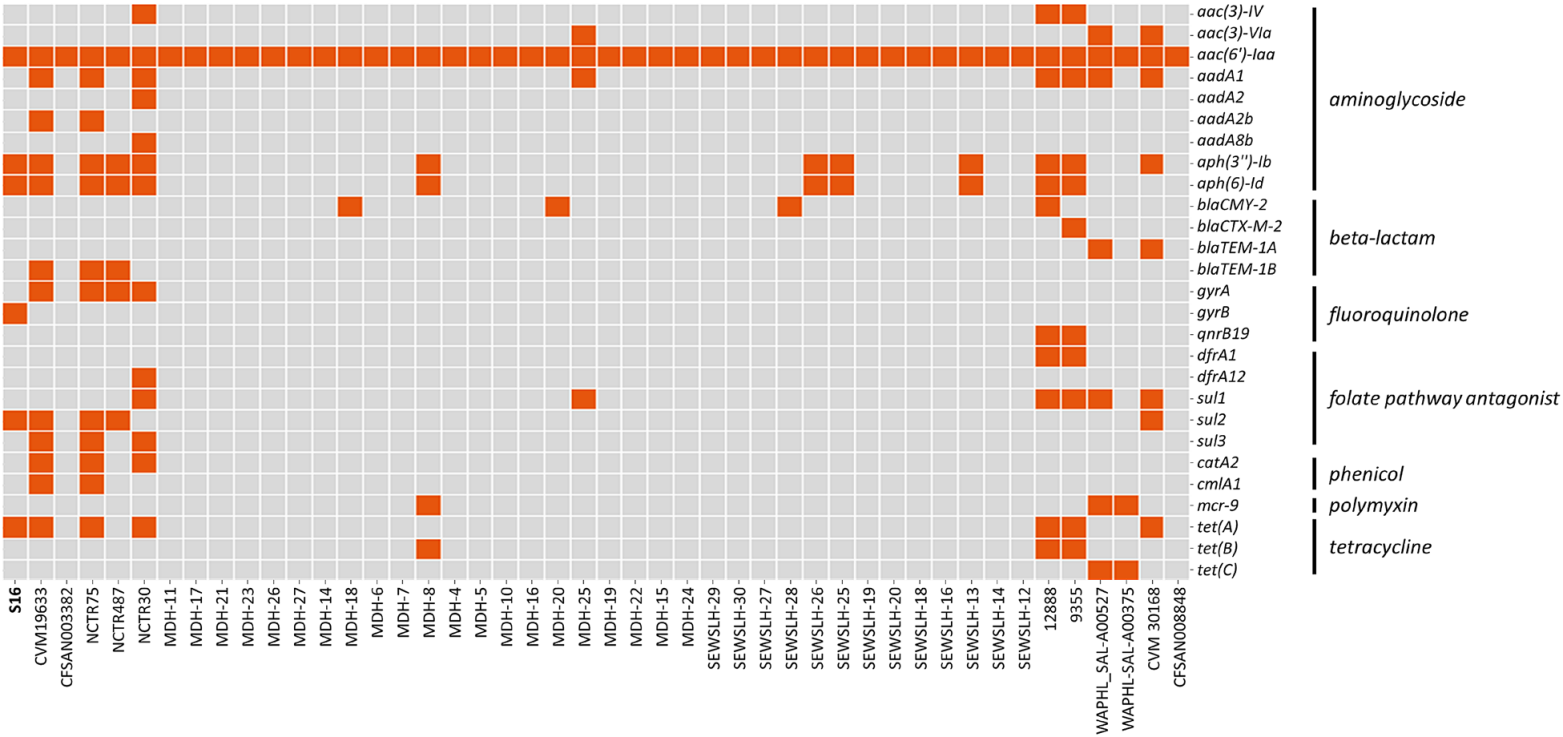
Heatmap of antibiotic resistance genes identified in the 46 *S*. Schwarzengrund genomes. Resistance genes are indicated as present (orange) or absent (gray). Row and columns represent resistance genes and each genome, respectively

Recent studies had found that *Salmonella* isolates exhibit the various profiles of multidrug resistance [34, 35]. The presence of antibiotic-resistance genes for 46 *S*. Schwarzengrund genomes were detected using the ResFinder database. Twenty-one different antibiotic-resistance genes were found in the 46 genomes (Fig. 3). All genomes carried at least one antibiotic-resistance gene. Additionally, they contained the *aac(6′)-Iaa* gene associated with resistance to aminoglycosides, consistent with previous studies [34, 36].

The strain S16 carries a multidrug-resistance profile against five antibiotics such as amikacin, streptomycin, ciprofloxacin, sulfamethoxazole, and tetracycline. In the MIC test, strain S16 showed resistance to FIS, STR, and TET, but not to AUG2, FOX, CHL, CIP, GEN, NAL, MEM, FEP, COL, SXT, AMP, CAZ, XNL, IMI, FOT, AXO, AZI, and AMK (Table 1). Genotype was concordant with broth microdilution, and genes responsible for resistance to sulfisoxazole, streptomycin, and tetracycline were found in the S16 genome. However, despite the presence of *gyrB* and *aac(6′)-Iaa* genes, strain S16 showed no resistance to ciprofloxacin and amikacin in MIC test. The *aac(6′)-Iaa* gene is responsible for resistance to amikacin, kanamycin, or tobramycin. Thus, strain S16 may have resistance to kanamycin or tobramycin, which was not tested in this study [37]. Also, the *gyrB* gene in the strain S16 genome may be related to resistance to other quinolone antibiotics, such as oxolinic acid, instead of ciprofloxacin [38].

**Table 1:** Comparison of antibiotic resistance genes and resistance phenotype for strain S16

Antimicrobial agents	MIC (mg/L)	Genetic resistance determinant
Amoxicillin/clavulanic acid (AUG2)	S or I (2)	–
Cefoxitin (FOX)	S or I (2)	–
Chloramphenicol (CHL)	S or I (8)	–
Ciprofloxacin (CIP)	S or I (0.12)	*gyrB*
Gentamycin (GEN)	S or I (1)	–
Tetracycline (TET)	R (128)	*tet(A)*
Nalidixic acid (NAL)	S or I (16)	–
Meropenem (MEM)	S or I (0.25)	–
Cefepime (FEP)	S or I (0.25)	–
Colistin (COL)	S or I (2)	
Trimethoprim/sulfamethoxazole (SXT)	S or I (0.25)	–
Ampicillin (AMP)	S or I (2)	–
Ceftazidime (CAZ)	S or I (1)	–
Sulfisoxazole (FIS)	R (512)	*sul2*
Ceftiofur (XNL)	S or I (1)	–
Streptomycin (STR)	R (256)	*aph(3″)-Ib*, *aph(6)-Id*
Imipenem (IMI)	S or I (1)	–
Cefotaxime (FOT)	S or I (1)	–
Ceftriaxone (AXO)	S or I (1)	–
Azithromycin (AZI)	S or I (4)	–
Amikacin (AMK)	S or I (4)	*aac(6′)-Iaa*

Among the resistance genes in the S16 genome, *aac(6′)-Iaa* gene was found to be present in the chromosome, whereas *aph(3″)-Ib*, *aph(6)-Id*, *sul2*, and *tetA* were found to be in the plasmid. Moreover, a single copy of *gyrB* gene was present on the chromosome. The strain S16 co-harbors the *tetA* gene, implicated in the resistance to tetracycline, and the *sul2* gene encoding sulfonamide-resistance. The *sul2* and *tetA* genes are the most prevalent resistance genes detected in animals and animal-food isolates [39, 40]. Quinolone (ciprofloxacin) is the antimicrobial drug of choice to treat complicated gastrointestinal infections, and resistance to ciprofloxacin has been reported in many isolates of *Salmonella* species [41–43]. Quinolone resistance in *Salmonella* serovars is often mediated by one or more mutations in DNA gyrase and topoisomerase IV [42]. Of the 46 *S.* Schwarzengrund genomes, five genomes, including strain S16, with a mutation in DNA gyrase were identified in this study. In clinical human and veterinary isolates of *Salmonella* serovars, mutations are usually confined to *gyrA* [41]. Similarly, mutations were observed at Ser-83 (Ser to Phe) and Asp-87 (Asp to Gly) of *gyrA* in CVM19633 and NCTR75 genomes; the mutation was observed only at Ser-83 (Ser to Phe) of *gyrA* in NCTR487 and NCTR30 genomes (Fig. 3). Unlike other genomes with *gyrA*, strain S16 showed a mutation at codon 464 of *gyrB* (Ser to Phe). Meanwhile, the *qnr* gene, a plasmid-mediated gene conferring resistance to antibiotics (such as ciprofloxacin) used to treat food animals [44], is also associated with quinolone resistance and was detected in two strains (12,888 and 9355) in this study.

### In silico identification of virulence genes

Virulence gene mapping of 46 genomes was performed to understand the virulence repertoire of *S*. Schwarzengrund. A total of 207 virulence genes were identified in the 46 genomes. A detailed result of virulence gene mapping for 46 *S*. Schwarzengrund genomes is shown in Additional file 1: Table S6. The virulence gene pattern of *S*. Schwarzengrund was similar to each strain, and a core set of virulence genes was conserved in all strains, regardless of geographic origin and isolation source. This result suggests that *S*. Schwarzengrund isolated from animal and environmental sources contained the same virulence factors as those carried by human clinical isolates. Additionally, several fimbrial genes and type III secretion systems 1 and 2, involved in cell invasion and survival of bacteria within phagocytes, were common to all *Salmonella* isolates [45]. These genes are also present in other serovars and are part of core genes with essential functions in *Salmonella* serovars [46].

In the strain S16 genome, 153 virulence genes, including fimbrial adherence determinants, non-fimbrial adherence determinants, macrophage-inducible genes, magnesium-uptake genes, regulation, secretion system, and toxin genes, were identified. *Salmonella* serovars contain multiple fimbriae on their surface, some of which play an essential role in adhesion to enterocytes to induce and maintain infection [47]. The *Salmonella* atypical fimbrial (*Saf*) operon, which is important for pathogenesis in serovars, such as Typhimurium, was absent in some serovars, such as Heidelberg, whereas it was present in all *S*. Schwarzengrund [47]. The cytolethal distending toxin *cdtB*, a typhoid-associated virulence factor, was detected in almost all genomes, including strain S16, similar to previous studies that detected this gene in *S*. Schwarzengrund isolates [40, 48]. Thus, these genomes harboring *cdtB* encode typhoid toxins that may contribute to human and animal pathogenicity.

## Conclusions

A comparative genomic analysis with the previously reported 45 genomes of *S.* Schwarzengrund provided a comprehensive analysis of the disease potential of strain S16. Comparative genomic analysis showed that the genomes of *S*. Schwarzengrund were similar regardless of geographic origin and isolate source, except that strain S16 had a mutation site for quinolone resistance gene that is different from the previously reported *S*. Schwarzengrund genomes. Our data will be a valuable resource to investigate nosocomial and foodborne infections.

## Supplementary Information

**Additional file 1: Table S1.** Summary in genome features of 45 Schwarzengrund. **Table S2.** Summary in IS-element and transposon in the S. Schwarzengrund strain S16. **Table S3.** Unique gene information of S. Schwarzengrund strain S16 obtained by pangenome analysis. **Table S4.** Genomic islands specific to strain S16. **Table S5.** List of unique genes present on the genomic islands. **Table S6.** Putative virulence genes in 46 *S.* Schwarzengrund genomes predicted by VFDB.

**Additional file 2: Figure S1.** The number of genes assigned in COG categories. Black and gray bars represent COGs of the *S*. Schwarzengrund strain S16 genome and unique genes of strain S16, respectively. **Figure S2.** Similarity graphical information indicating whole-genome sequence identity of strain S16 genome with reference genome (*S*. Schwarzengrund CVM19633). Gray arrows in the figure indicate the orientation of genes. A cut-off of 50% identity was used. The y-scale axis indicates the identity within 50–100%. **Figure S3.** Arrow diagrams for the five genomic islands specific to strain S16. Genes and their orientation are shown with arrows; green, blue, red, and gray indicate known proteins, transposase, mobile element proteins, and hypothetical proteins.

## Data Availability

The complete genome data of *Salmonella enterica* subsp. *enterica* serovar Schwarzengrund strain S16 has been deposited in DDBJ/EMBL/GenBank, with Accession Number CP081858.1-CP081859.1.
