# Supplementary material for: Genomic characteristics and comparative genomics of Salmonella enterica subsp. enterica serovar Schwarzengrund strain S16 isolated from chicken feces
Source: Gut Pathog. 2022 Jan 4;14:1. doi: 10.1186/s13099-021-00476-8 (PMC8728987; doi:10.1186/s13099-021-00476-8)
Supplement: Supplementary file 2 — Additional file 2: Figure S1. The number of genes assigned in COG categories. Black and gray bars represent COGs of the S. Schwarzengrund strain S16 genome and unique genes of strain S16, respectively. Figure S2. Similarity graphical information indicating whole-genome sequence identity of strain S16 genome with reference genome (S. Schwarzengrund CVM19633). Gray arrows in the figure indicate the orientation of genes. A cut-off of 50% identity was used. The y-scale axis indicates the identity within 50–100%. Figure S3. Arrow diagrams for the five genomic islands specific to strain S16. Genes and their orientation are shown with arrows; green, blue, red, and gray indicate known proteins, transposase, mobile element proteins, and hypothetical proteins. [file 13099_2021_476_MOESM2_ESM.docx]

***Supplementary Materials***

**
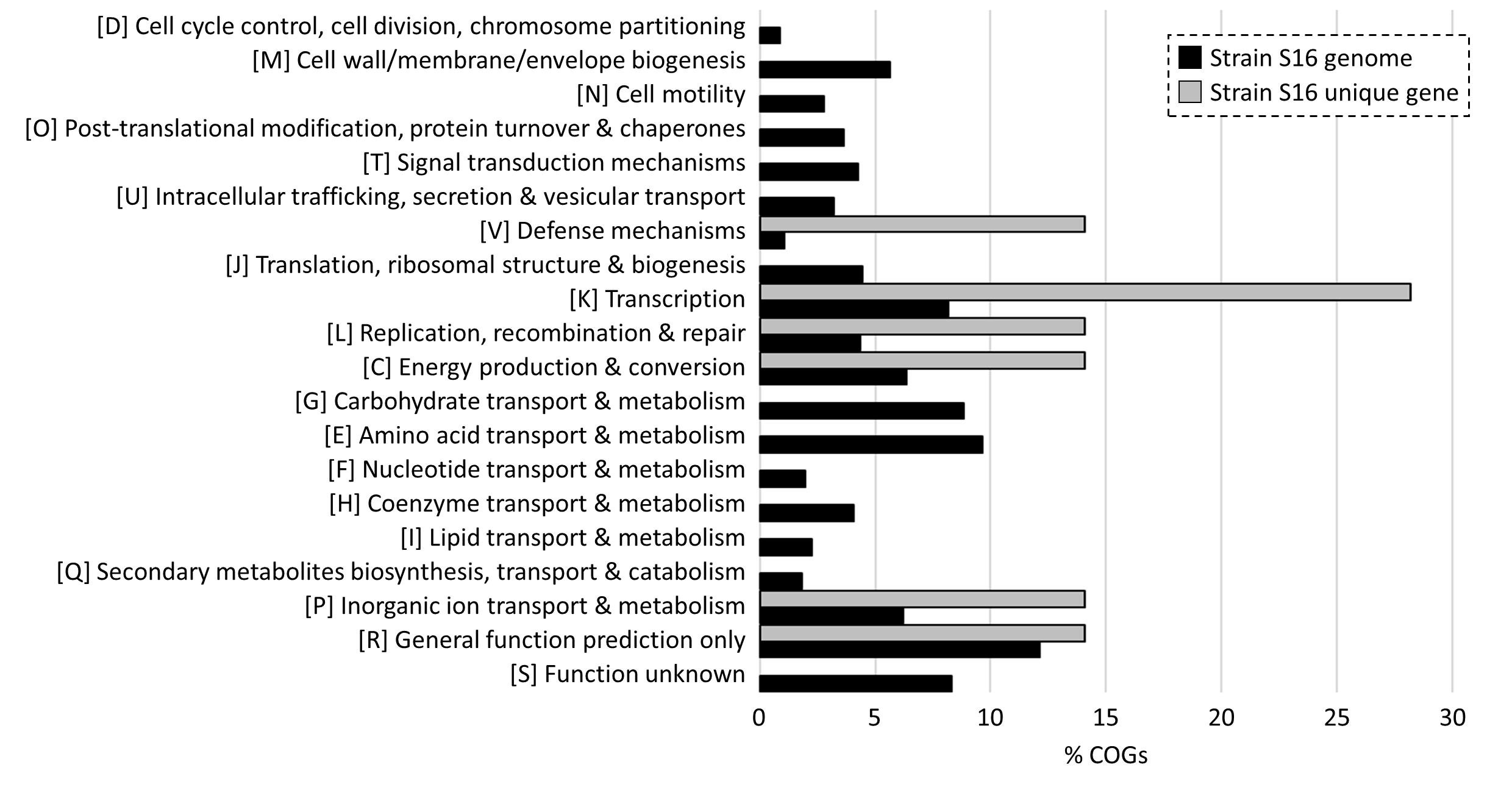
**

**Fig. S1.** The number of genes assigned in COG categories. Black and gray bars represent COGs of the *S*. Schwarzengrund strain S16 genome and unique genes of strain S16, respectively.

**
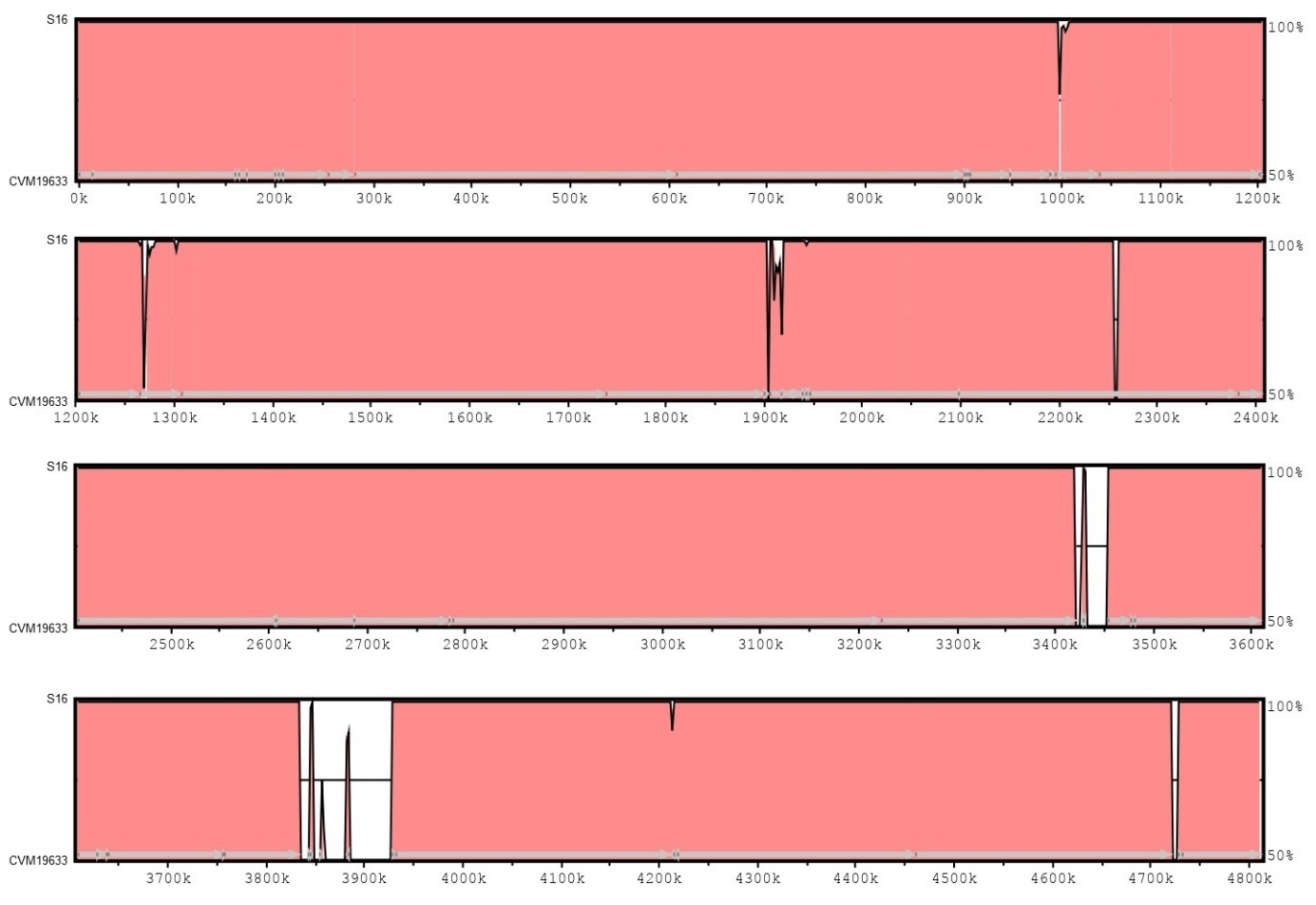
**

**Fig. S2.** Similarity graphical information indicating whole-genome sequence identity of strain S16 genome with reference genome (*S*. Schwarzengrund CVM19633). Gray arrows in the figure indicate the orientation of genes. A cut-off of 50% identity was used. The y-scale axis indicates the identity within 50%-100%.

**
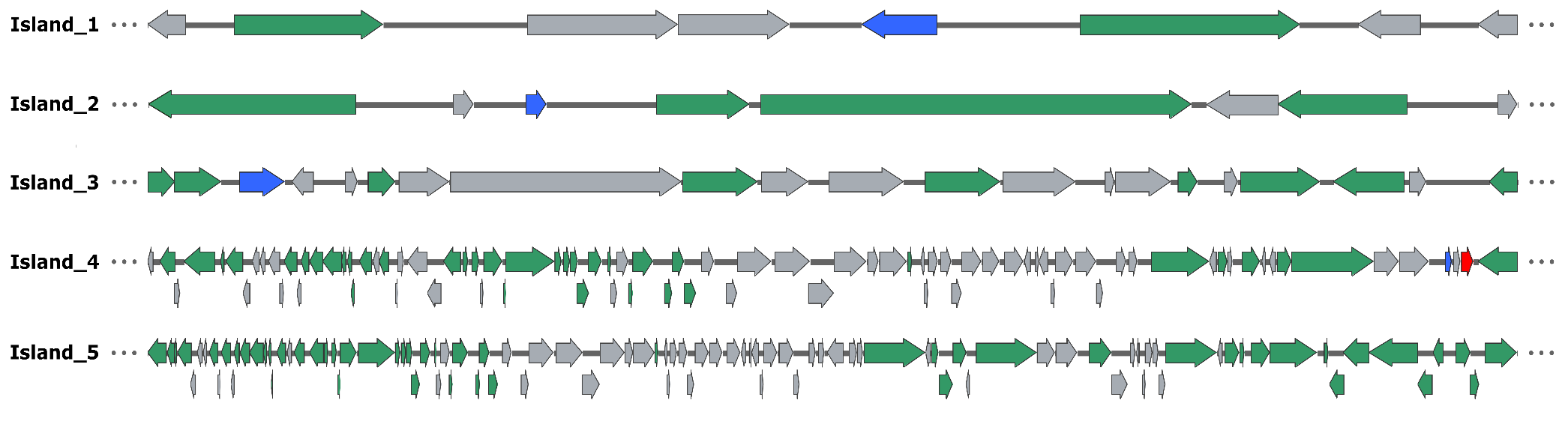
**

**Fig. S3.** Arrow diagrams for the five genomic islands specific to strain S16. Genes and their orientation are shown with arrows; green, blue, red, and gray indicate known proteins, transposase, mobile element proteins, and hypothetical proteins.
